# Causal relationship between the gut microbiota, immune cells, and coronary heart disease: a mediated Mendelian randomization analysis

**DOI:** 10.3389/fmicb.2024.1449935

**Published:** 2024-08-05

**Authors:** Feifei Yang, Hui Song, Weizhi Tang, Lingyun Liu, Ziyi Zhu, Bin Ouyang, Liwen Zhang, Guixin He, Weibin Qin

**Affiliations:** ^1^Graduate School of Guangxi University of Chinese Medicine, Nanning, China; ^2^Department of Cardiovascular Medicine, The First Affiliated Hospital of Guangxi University of Chinese Medicine, Nanning, China

**Keywords:** gut microbiota, immune cells, coronary heart disease, Mendelian randomization, genome-wide association studies

## Abstract

**Background:**

Recent studies have shown that the gut microbiota (GM), immune cells, and coronary heart disease (CHD) are closely related, but the causal nature of these relationships is largely unknown. This study aimed to investigate this causal relationship and reveal the effect of GM and immune cells on the risk of developing CHD using mediated Mendelian randomization (MR) analysis.

**Methods:**

First, we searched for data related to GM, immune cells, and CHD through published genome-wide association studies (GWAS). We filtered the single nucleotide polymorphisms (SNPs) associated with GM and immune cells and then performed the first MR analysis to identify disease-associated intestinal bacteria and disease-associated immune cells. Subsequently, three MR analyses were conducted: from disease-associated GM to disease-associated immune cells, from disease-associated immune cells to CHD, and from disease-associated GM to CHD. Each MR analysis was conducted using inverse variance weighting (IVW), MR-Egger regression, weighted median, weighted models, and simple models.

**Results:**

A total of six GM and 25 immune cells were found to be associated with CHD. In the MR analysis using the inverse variance weighting (IVW) method, g__Desulfovibrio.s__Desulfovibrio_piger was associated with EM DN (CD4–CD8–) %T cells (*P* < 0.05 and OR > 1), EM DN (CD4–CD8–) %T cells was associated with CHD (*P* < 0.05 and OR < 1), and g__Desulfovibrio.s__Desulfovibrio_piger was associated with CHD (*P* < 0.05 and OR < 1).

**Conclusion:**

An increase in the abundance of g__Desulfovibrio.s__Desulfovibrio_piger leads to an increase in the amount of EM DN (CD4–CD8–) %T cells, and an increase in the amount of EM DN (CD4–CD8–) %T cells reduces the risk of developing CHD. Our study provides some references for reducing the incidence of CHD by regulating GM and immune cells.

## 1 Introduction

Cardiovascular disease is the most common cause of death worldwide. Coronary heart disease (CHD) is the most common type of cardiovascular disease. It is characterized by dysfunction of the heart muscle due to the narrowing of the coronary arteries, which results in inadequate blood supply. CHD is one of the leading causes of death in elderly patients worldwide, and its risk continues to rise. In mouse models, susceptibility to CHD and thrombosis can be transmitted through gut microbial (GM) transfer (Brown and Hazen, 2018). This transmission may be related to the fact that microbial communities influence host metabolism and are sensed by host pattern-recognition receptors through microbe-associated molecular patterns, which influence the pathogenesis of cardiovascular disease. Microbe-targeted therapeutic strategies are expected to prevent or treat cardiovascular diseases (Brown and Hazen, 2018). There are significant differences in the composition of the GM between normal individuals and patients with coronary artery disease. In healthy people, the intestinal microbiota mainly comprises Firmicutes, Bacteroidetes, Actinobacteria, and Ascomycetes, which play a key role in maintaining intestinal health and the immune system.

In contrast, patients with CHD experience significant changes in the composition and structure of their gut flora. These changes include increases or decreases in certain bacterial groups, such as *Trichoderma spiraceae* Lachnospiraceae and Ruminococcaceae, as well as an increase in the number of pathogens or opportunistic pathogens (Dai et al., 2020). Potential biomarkers reported to date include trimethylamine oxide (TMAO), short-chain fatty acids (SCFA), and secondary bile acids. For example, TMAO is an intestinal flora metabolite that is strongly associated with atherosclerosis formation and the development of CHD. Studies have shown that TMAO promotes atherosclerosis formation by affecting platelet activity and cholesterol metabolism (Tang and Hazen, 2017; Witkowski et al., 2020).

Previous studies have shown a strong causal relationship between GM and CHD (Jiang et al., 2023; Yang et al., 2024), and changes in the abundance of GM and metabolites may influence the progression of CHD (Wang et al., 2024). A large body of evidence suggests that GM plays a crucial role in the onset and progression of diseases such as metabolic disorders and cardiovascular diseases (Wen et al., 2022; Qiao et al., 2023). Clinical studies have found significant changes in GM in patients with CHD and cognitive impairment (Sun et al., 2019; Paiva et al., 2020). Changes in GM can mediate the development of CHD through mechanisms such as chronic inflammation, promoting atherosclerosis, and facilitating thrombosis (Liyu et al., 2022).

One study analyzed the relationship between the GM and CHD from the perspective of the transcriptome and found that Fusicatenibacter can be highly correlated by affecting several CHD-related targets, namely GBP2, MLKL, and CPR65 (Chen et al., 2023). Another study suggested a sex-specific dysbiosis in the intestinal microbiota linked to CHD, potentially contributing to the sex disparity observed in cardiovascular disease incidence (Garcia-Fernandez et al., 2024). Many herbs could also act on the GM to intervene in CHD by modulating the composition of the GM, reducing trimethylamine-N-oxide (TMAO) levels, increasing short-chain fatty acids (SCFAs), and maintaining appropriate bile acid (BA) levels (Cao et al., 2024).

It is possible that gut fungus (an important component of the GM) can serve as a biomarker to differentiate between the different stages in the development and progression of CHD and facilitate the development of therapeutic targets. However, there are significant challenges, such as the influence of hypertension, diabetes, and smoking, as well as other potential confounders, in using GM as a marker for CHD. These confounding factors make it difficult to identify specific GM components causally linked to CHD amid other comorbid disease clusters.

One of the characteristics of CHD is chronic inflammation, especially where atherosclerotic plaque accumulation contains immune cytokines in various states of activation and differentiation, including CD4+ helper T-cells, CD8+ killer T cells, macrophages, and natural killer cells (NK cells) that secrete inflammatory mediator (Fernandez et al., 2019; Depuydt et al., 2020). A single-cell technology assay study showed that the immune microenvironment of human coronary atherosclerotic plaques at different stages of atherosclerosis revealed a high proportion of αβ T cells and predominantly memory cells that experience antigen (Chowdhury et al., 2022). Without active infection and latent autoimmune-mediated T cell activation, T cells in plaques may undergo clonal expansion through antigen binding and are potentially reactive to their epitopes (Chowdhury et al., 2022). However, the interaction of immune cells with CHD is unclear. Studying how these immune cells contribute to CHD progression may facilitate the development of new therapeutic strategies.

GM can modulate immune system responses by influencing the differentiation and function of immune cells. For example, it has been found that specific intestinal bacteria can convert dietary linoleic acid to conjugated linoleic acid (CLA) through fatty acid isomerization, which, in turn, affects intestinal mucosal immunity (Song et al., 2023). GM not only affects the immune response in the intestine but also participates in the occurrence and development of extraintestinal diseases by regulating immune cells. Intestinal flora and their metabolites can participate in the occurrence of various extraintestinal diseases, such as cancer, cardiovascular diseases, autoimmune diseases, and so on, by affecting the migration and function of immune cells (Jiahao et al., 2024). For example, GM may enhance the therapeutic efficacy of immune checkpoint inhibitors (ICIs) and reduce the side effects of ICI therapy. Diet, prebiotics, probiotics, and fecal transplants can improve the GM and thus improve ICI efficacy (Simpson et al., 2023).

Mendelian randomization (MR) is an efficient and rigorous method for inferring causality that employs genetic variation as an instrumental variable (IV) to explore the causal effects of etiologically inferred exposures on outcomes (Emdin et al., 2017). This approach minimizes the impact of confounding factors on causal estimates while avoiding reverse causation through Mendelian laws of inheritance. MR plays an important role in understanding treatments for the etiology of cardiovascular diseases (Emdin et al., 2017; Levin and Burgess, 2024). In this study, we chose GM as the exposure factor, immune cells as the mediator, and CHD as the outcome variable. We then used two-step Mendelian randomization (TSMR) to explore further the potential causal relationships between GM, immune cells, and CHD.

## 2 Materials and methods

### 2.1 Study design

**Figure 1:**
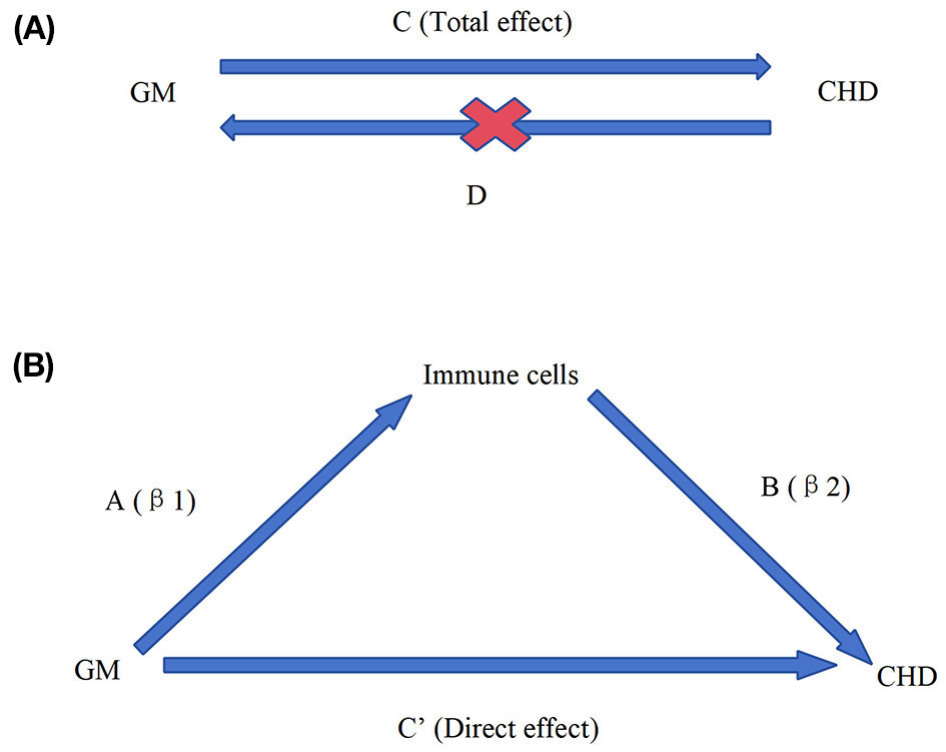
Overall design ideas for this study. Diagrams illustrating associations were examined in this study. **(A)** The total effect between gut microbiota (GM) and coronary heart disease (CHD). C refers to the total effect using genetically predicted GM as the exposure and CHD as the outcome. D refers to the total effect of genetically predicted CHD exposure and GM as the outcome. **(B)** The total effect was decomposed into (i) the indirect effect using a two-step approach (where a is the total effect of the GM on immune cells and b is the effect of immune cells on CHD and the product method (a × b); (ii) the direct effect (c′ = c – a × b). Proportion mediated was the indirect effect divided by the total effect.

We performed a two-step MR analysis to explore the mediating role of immune cells in the relationship between GM and CHD. All the data used in the analysis were obtained from publicly accessible GWAS. We extracted single nucleotide polymorphisms (SNPs) associated with gut microbial taxa and immune cells, which were then used as instrumental variables (IV). We performed the mediated MR analysis using pooled data from GWAS for GM, immune cells, and CHD. The design concept of this study is shown in Figure 1.

### 2.2 Screening for disease-associated GM and disease-associated immune cells

First, GM exposure data were collected. Summary statistic data for GM were obtained from a genome-wide association study (GWAS) of 207 intestinal microbial taxa (g phyla, 10 classes, 13 orders, 26 families, 48 genera, and 105 species) conducted by the Dutch Microbiome Project. Data at the genus level were collected from a specific database (http://ftp.ebi.ac.uk/pub/databases/gwas/summary_statistics/), which included 412 genera (GCST90027446 to GCST90027857) with a sample size of 7,738 (Lopera-Maya et al., 2022).

Subsequently, immune cell exposure data were obtained. A total of 731 immune cell datasets (GCST90001391 to GCST90002121) were acquired through the GWAS Catalog database (https://www.ebi.ac.uk/gwas/), with a sample size of 5,959. The acquired GM and immune cell data were then filtered. The SNPs associated with intestinal flora and immune cells could be found by setting the *p-*value to < 1e-05 for the association analysis (Lv et al., 2021).

Linkage disequilibrium (LD) refers to the non-random association between neighboring genes or genetic markers located in the genome. Two parameters, kilobases (kb) and linkage disequilibrium coefficient (r^2^), are used to describe the degree and extent of linkage disequilibrium. If two SNPs are close to each other, one of them must be removed, and LD SNPs were removed by setting the distance parameters to kb = 10,000 and r^2^ = 0.001, retaining only the SNP with the highest significance for subsequent analysis.

Weak instrumental variables, which are not strongly correlated with the exposure factors or only explain a small fraction of the phenotypic variance, were removed by setting a test value of *F* > 10 (Yengo et al., 2018). The remaining SNPs, which were strongly correlated with the GM and immune cells, were used as IVs for the subsequent Mendelian randomization analysis. Finally, genomic data related to CHD were obtained through the IEU Open GWAS Project database (https://gwas.mrcieu.ac.uk/). The sample size of CHD included in this study was 86,995, with a total of 2,420,361 SNPs.

Performing an MR analysis of GM in relation to CHD reveals which GM types are causally related to CHD. By filtering these results, we can pinpoint the GM associated with the disease. Additionally, a reverse MR analysis was conducted on the disease-related intestinal flora. If this reverse analysis yields negative results, a subsequent MR analysis could be considered. Furthermore, an MR analysis of immune cells specific to CHD was conducted to determine the causal relationship between specific immune cells and the disease. Filtering the results of this analysis helps identify immune cells associated with CHD.

### 2.3 Mediated Mendelian randomization analysis

A mediated MR analysis was conducted after identifying CHD-associated GM and CHD-associated immune cells. An MR analysis between disease-associated GM and disease-associated immune cells was conducted to determine the β1 value, representing the effect of immune cells on the disease. Subsequently, an MR analysis was conducted to ascertain the β2 value, reflecting the influence of disease-associated GM on the disease. The total effect value was then calculated using MR analysis. Finally, the mediated effect value, calculated as the product of β1 and β2, quantifies the extent to which intestinal flora impacts CHD by influencing specific immune cells that act as mediators (Cai et al., 2022). All analyses were conducted using R software version 4.4.0.

### 2.4 Statistical analysis

The main analytical method for MR was the inverse variance weighted (IVW) method (Xin et al., 2022), which is considered the most accurate and robust method. In addition, we used four supplementary MR methods to bolster the reliability of our results: MR-Egger (Burgess and Thompson, 2017), weighted median (Bowden et al., 2016), simple mode (Xiang et al., 2022), and weighted mode (Xiang et al., 2022). If the β value from the IVW method is >0, it indicates that the exposure factor is a risk factor. On the contrary, if the β value is < 0, it suggests that the exposure factor is a protective factor. If the *p*-value of the IVW method is < 0.05, it implies that there is a causal relationship between the exposure factor and the outcome, whereas if the *p-*value is >0.05, it indicates no causal relationship exists between the exposure factor and the outcome. Similarly, an odds ratio (OR) >1 from the IVW method suggests that the exposure factor is a risk factor, while an OR < 1 indicates that it is a protective factor. We also applied multiple corrections to significant results based on specific conditions to ensure the utmost reliability of our findings.

## 3 Results

### 3.1 Selection of instrumental variables

After obtaining the exposure data for GM from public databases, we identified 4,031 SNPs associated with GM (Supplementary Table 1) and 14,696 SNPs associated with immune cells (Supplementary Table 2) as instrumental variables by setting a significance threshold of *P* < 1 × 10^−5^. We excluded weak instrumental variables associated with both GM and immune cells by setting an F-statistic threshold >10. Consequently, we retained 1,903 strong instrumental variables associated with GM (Supplementary Table 3) and 14,696 variables associated with immune cells (Supplementary Table 4), representing genetic variants with high explanatory power for GM and immune cells, respectively. In the above results, we systematically provide detailed information on the key features of these SNPs, including effector alleles, other alleles, β values, *p*-values, standard error values, R2 values, F-values, and so on, to support further analysis in subsequent studies.

### 3.2 Identification of disease-associated GM and disease-associated immune cells

The MR analysis of GM to CHD yielded data on 928 results (Supplementary Table 5). We filtered these results using the inverse variance weighted (IVW) method with a significance threshold of *P* < 0.05 to screen for six disease-associated GM: the families Clostridiaceae and Sutterellaceae, the genus *Clostridium*, and *Escherichia* unclassified species (Supplementary Table 6). Additionally, a reverse MR analysis performed on these disease-associated GM, with *P-*values >0.05 for all six GM using the IVW method (Supplementary Table 7), yielded negative results from the reverse analysis. This allowed for the progression of subsequent mediated MR analyses.

### 3.3 Mediated Mendelian randomization analysis

After identifying the disease-associated GM and disease-associated immune cells, we were able to conduct three mediated MR analyses. The first MR analysis, conducted from disease-associated GM to disease-associated immune cells, yielded a β1 value of 0.147, indicating a positive association (Supplementary Table 10). This analysis included MR-Egger and four sensitivity analyses, all confirming significance with a *p-*value of < 0.05 using the IVW method.

The second MR analysis was conducted from disease-associated immune cells to the disease, resulting in a β2 value of −0.120, suggesting a protective effect. Relevant data regarding MR-Egger and four sensitivity analyses are detailed in Supplementary Table 11, with all results showing a *p*-value of < 0.05 using the IVW method.

The third analysis evaluated the relationship between disease-associated intestinal bacteria and the disease. It provided a total effect β value of −0.138, with relevant data regarding MR-EGGER and four sensitivity analyses detailed in Supplementary Table 12. Despite the significance found in the IVW method (*P* < 0.05), the MR-PRESSO method yielded a *p-*value of more than 0.05 across all SNPs (*P* > 0.05), indicating no combined outliers for all SNPs. The calculated mediating effect, β1 × β2, was −0.0176, with a range of −0.0452 to 0.0099. This suggests that GM from g__Desulfovibrio.s__Desulfovibrio_piger may act as mediators of CHD through the modulation of EM DN (CD4–CD8–) %T cells, a specific immune cell type (Supplementary Table 13). This provides insight into the potential pathway through which GM influences CHD.

**Figure 2:**
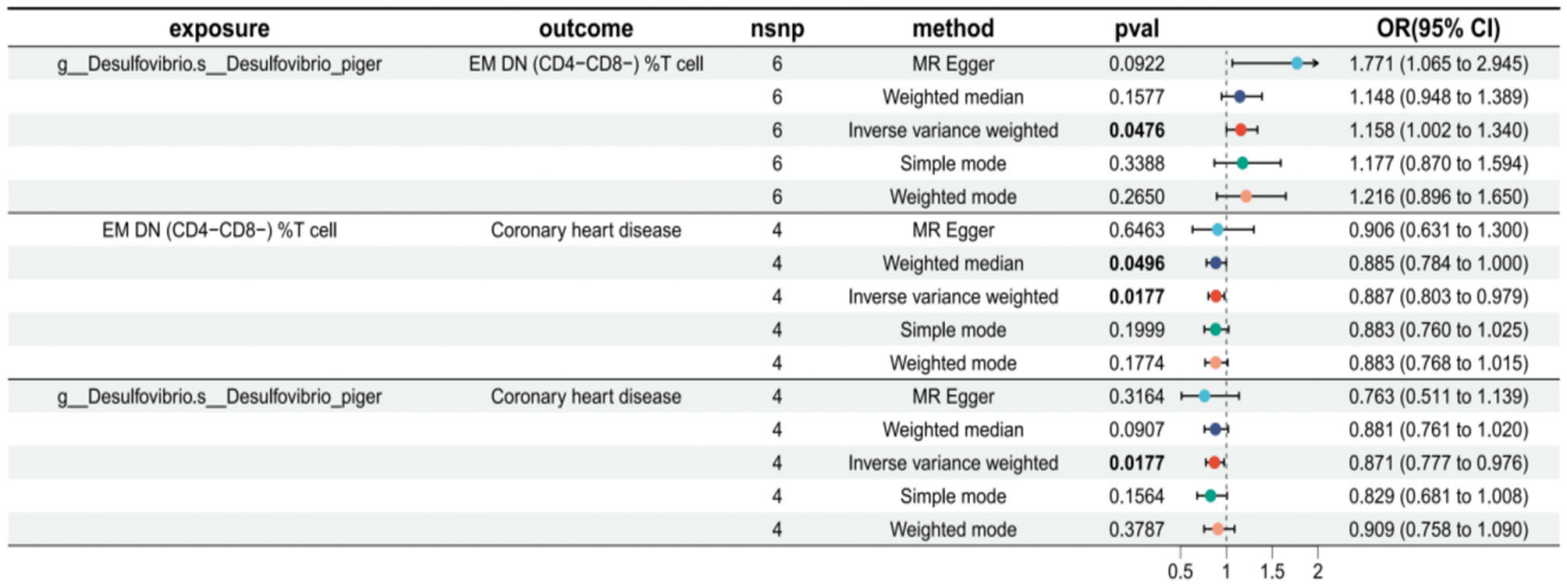
The relationship between exposure, mediator, and disease.

In addition, the visualized forest plot from the mediator MR analysis (Figure 2) clearly shows the relationship between exposure, mediator, and disease. The *p*-values from the IVW method in all three MR analyses were below 0.05, indicating statistically significant results across all three MRs. In the first MR analysis, OR was >1 (95% CI: 1.002 to 1.340), suggesting that the gut microbiota species g__Desulfovibrio.s__Desulfovibrio_piger, an intestinal bacterium, is a risk factor. Specifically, an increase in this bacterium correlates with an increase in the EM DN (CD4–CD8–) %T cells, indicating that higher levels of this intestinal bacterium enhance the presence of these immune cells. Conversely, the second MR analysis showed an OR < 1 (95% CI: 0.803 to 0.979), indicating that the EM DN (CD4–CD8–) %T cells act as a protective factor against disease development; as the levels of these immune cells increase, the risk of CHD decreases.

The third MR analysis also reported an OR < 1 (95% CI: 0.777 to 0.976), confirming that the presence of g__Desulfovibrio.s__Desulfovibrio_piger acts as a protective factor. An increase in this bacterial species corresponds with a decreased risk of developing CHD. These findings suggest that higher levels of g__Desulfovibrio.s__Desulfovibrio_piger not only increase the beneficial EM DN (CD4–CD8–) %T cells but also contribute directly to reducing the risk of CHD.

## 4 Discussion

**Figure 3:**
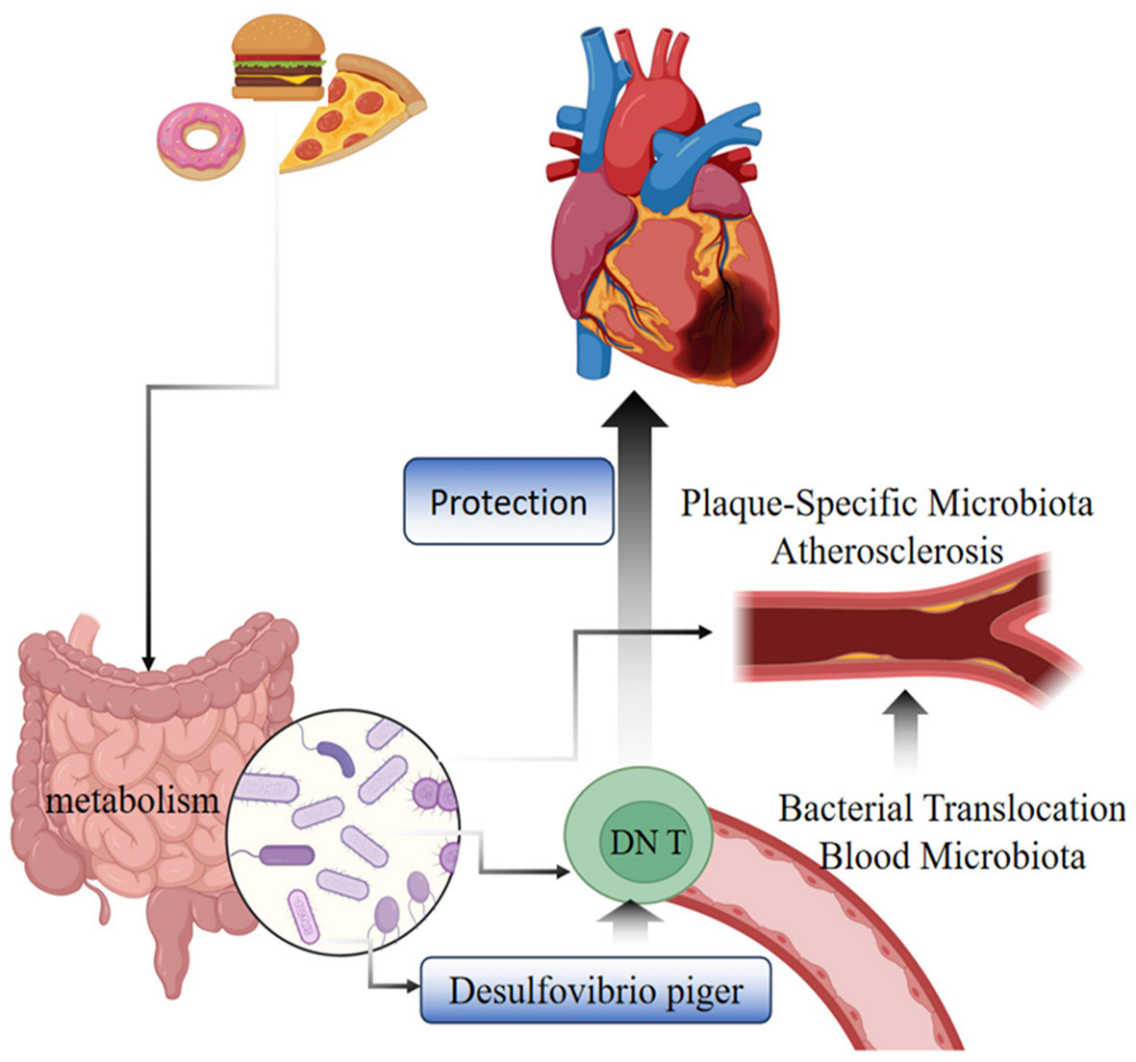
Diagram of the mechanism of CHD protection by g__Desulfovibrio.s__Desulfovibrio_piger bacteria through EM DNT cells.

Our study is the first MR study to investigate the causal relationship between GM, immune cells, and CHD, offering critical insights into the pathogenic mechanisms of the disease and aiding in the development of effective prevention and treatment strategies. Utilizing pooled data from genome-wide association studies (GWAS), we conducted a two-step MR with mediation analysis to explore the potential causal relationships between GM, immune cells, and CHD. Observed in a European population, our findings reveal that, using genetic variation as a diagnostic tool in 5 distinct MR analyses, 6 specific gut microbial taxa and 25 immune cells were causally associated with CHD. Notably, our mediated MR study identified g__Desulfovibrio.s__Desulfovibrio_piger as being protective against CHD through its influence on EM DN (CD4–CD8–) %T cells, a mechanism shown in Figure 3.

Dysbiosis of GM is associated with nearly 95% of health conditions, especially cardiometabolic diseases, including CHD (Fan and Pedersen, 2021). Increasing collaborative efforts to deeply understand the diversity of the human gut microbiome are prompting the development of more precise and personalized approaches to disease prevention (Rahman et al., 2022; Chakaroun et al., 2023).

A two-sample Mendelian study found that *Desulfovibrio* spp. and *A. tumefaciens* UCG010 exhibit protective effects against coronary atherosclerosis (Zhao et al., 2022). Although *Desulfovibrio*, a dual-action bacterium, is associated with several diseases, it has shown positive associations with beneficial microbial genera and negative associations with harmful ones (Chen et al., 2021). At the genus level, *Desulfovibrio* correlates negatively with CHD risk factors such as body mass index (BMI), waist circumference, triglycerides (TG), and uric acid (UA), while its relative abundance positively correlates with the diversity of health-associated microbial communities (Vieira-Silva et al., 2016; Chen et al., 2021). In addition, experimental studies have identified a protective role of *Desulfovibrio* in a myriad of cardiovascular diseases such as myocardial ischemia-reperfusion injury, potentially due to its effects on reducing the production and circulation of lipopolysaccharide (LPS), thereby inhibiting circulatory and inflammation-related cardiovascular diseases (Bai et al., 2021). Levels of *Desulfovibrio* are significantly reduced in patients with coronary artery disease coexisting with type 2 diabetes mellitus (CHD-DM2) (Sanchez-Alcoholado et al., 2017).

Desulfovibrio piger is a widespread sulfate-reducing bacterium in the human gut, known for producing substances such as hydrogen sulfide through sulfate reduction (Warren et al., 2005; Olsson et al., 2022). This bacterium, along with other sulfate-reducing bacteria, helps animals to digest indigestible substances such as cellulose and produces beneficial metabolites (Nirmalkar et al., 2022). The relationship between gut microbiota and metabolic regulation is further illustrated by studies on diabetes mellitus, which is closely associated with the development of coronary heart disease (CHD). In type 2 diabetes mellitus (T2DM), hydrogen sulfide and sulfate-based prebiotics have been shown to stimulate the secretion of glucagon-like peptide-1 (GLP-1), thereby improving blood glucose levels in male mice. This provides insights into how gastrointestinal microbes and their metabolites regulate the metabolism (Pichette et al., 2017). Additionally, research indicates that Desulfovibrio piger can enter the plasma and produce alkylglycerol phosphate (A-GPC), which inhibits CXCR3+ T-cell activity and thus potentially mitigates the autoimmunogenesis of type 1 diabetes (T1D), highlighting a significant immunological interaction (de Groot et al., 2021). It has also been pointed out that modulating specific gut flora to increase or decrease various types of T cells can expedite the recovery of renal function in severe ischemic acute kidney injury (Gharaie et al., 2023).

A growing body of evidence suggests that changes in microbiota composition and diversity can promote human health by modulating the immune-inflammatory response (Ansaldo et al., 2021). Our results suggested that g__Desulfovibrio.s__Desulfovibrio_piger acts as a protective factor against CHD by increasing the presence of EM DN (CD4–CD8–) %T cells. Further supporting this finding, analysis of the bacterial flora in long-lived older adults (over 100 years) showed an increase in flora diversity (GBD 2019 Diseases and Injuries Collaborators, 2020). This diversity is associated with elevated secretion of the anti-inflammatory cytokine IL-10 and the presence of several potentially probiotic species, including g__Desulfovibrio.s__Desulfovibrio_piger, *Gordonibacter pamelae*, and *Odoribacter splanchnicus*. These changes may contribute to health maintenance and longevity (Wang et al., 2022).

The generation and maintenance of immune memory are essential for maintaining a protective immune response capable of controlling pathogens and performing immune surveillance (Ahmed and Gray, 1996). After their initial exposure to a specific antigen, naïve cells differentiate into two main cellular subpopulations: central memory cells and effector memory (EM) cells (Sallusto et al., 1999). Effector memory T (T_EM_) cells are mainly found in the peripheral areas, such as the skin, mucous membranes, and perivascular areas, where they are formed after the first encounter with a specific antigen and rapidly exert an immune response when the same antigen is encountered again (Butcher and Picker, 1996). T_EM_ cells have been found to promote atherosclerosis (Ammirati et al., 2015).

A single-cell, high-dimensional analysis of peripheral blood from humans with coronary artery disease highlighted predominantly significant cell-to-cell interactions between CD4 effector/memory cells and the intermediate monocyte subpopulation (iMo) (Chatterjee et al., 2024).

We believe that CD4+ and CD8+ T cells play an important role in inflammation, which is a core pathological process in atherosclerosis (Zhou et al., 2000; Hansson and Hermansson, 2011; Wang et al., 2023).

Our study focuses on CD4– and CD8–double-negative (DN) T cells, which are present in low amounts, which constitute 1–3% of human T cells and exhibit diverse origins and development pathways in peripheral tissues (Fischer et al., 2005; Newman-Rivera et al., 2022). These cells, lacking CD4 and CD8 co-receptors, can endure chronic stimuli, positioning them as key players in chronic inflammation immunity (Hamad et al., 2001; Wu et al., 2022). DNT cells are present in several organs, including the kidney, lungs, heart, gastrointestinal tract, and liver (Anders et al., 2020), and their therapeutic applications have shown promise in cardiac graft-vs.-host disease survival and autoimmune myocarditis (Chen et al., 2005). In specific inflammatory conditions such as Chagas cardiomyopathy (Chagas cardiomyopathy), targeting the activation of EM cells and DN T cells may offer a strategic approach to managing the disease (Passos et al., 2021). This protective effect contrasts sharply with the destructive role of CD4+CD8+ T cells in CHD.

Although previous observational studies have identified associations between immune cells and CHD (Wang et al., 2023), they often face limitations such as the inability to eliminate potential confounders, selection bias, and small sample sizes. Additionally, the costly and time-consuming nature of randomized controlled trials has posed a challenge in establishing causality between gut microbes, immune cells, and CHD. To overcome these challenges, we utilized summary statistics from recent large-scale genomic and immune cell profiling studies to provide theoretical support for the associations noted in earlier observational studies. Our findings were robust, with no multicollinearity or heterogeneity detected in the sensitivity analyses. Exploring the genetic associations between gut microbiota (GM), immune cells, and CHD could offer new avenues for early diagnosis, using these elements as biomarkers for CHD, and developing more effective treatments. This study highlights the importance of investigating the mechanistic associations between GM, immune cells, and CHD at the genetic level.

The study of genes has gained significant attention in recent years, particularly with the advancements in single-cell RNA sequencing (scRNA-seq), which have accelerated research progress. For instance, a previous study utilized immune cell scRNA-seq data integrated with genome-wide association studies (GWAS) on COVID-19 to identify crucial immune cell types associated with severe cases of the disease (Ma et al., 2022). This approach offers valuable insights into studying the relationship between immune cells and coronary heart disease (CHD). However, despite its potential, scRNA-seq technology is expensive, and the typically small sample sizes of single-cell data lack sufficient statistical power to identify phenotype-related cell subsets.

Addressing these challenges, a high-quality research team has developed the scPagwas method, which synergistically combines scRNA-seq data with large-sample GWAS data. This innovative method allows for the identification of disease-associated cell subsets at single-cell resolution levels (Ma et al., 2023). In our upcoming research, we plan to employ the scPagwas method to integrate CHD/GM GWAS data with immune cell scRNA-seq data. This will enable us to identify CHD-associated immune cells and uncover critical risk genes and pathways that may play a role in the causal relationship between GM and CHD.

Unlike previous MR analyses, our study used mediated Mendelian randomization analysis, which synergistically combines traditional MR with mediation analysis. This method investigates the causal effects of an exposure factor on an outcome through a mediating variable (Peng et al., 2020). One of its main benefits is that it enables a pure causal inference, providing a clear understanding of the pathways involved (Sanderson, 2021). In our study, we analyzed the causal effect of GM on immune cells and the subsequent causal impact of these immune cells on coronary heart disease (CHD). With GM serving as the exposure factor, immune cells as the mediator, and CHD as the outcome, our findings demonstrated that an increase in the bacterium g__Desulfovibrio.s__Desulfovibrio_piger simultaneously increased the levels of EM DN (CD4–CD8–) %T cells, which, in turn, contributed to a reduced risk of CHD.

We made multiple corrections to account for significant results; however, the corrected *p*-values have not been included in the article due to their not meeting the threshold for inclusion indicated in the Results section. This pertains specifically to the *p*-values obtained from disease-related intestinal bacteria and the filtration of immune cells using the IVW method and the *p*-values for pleiotropy.

Our study boasts several strengths. First, it utilizes data from the largest sample size in GWAS, ensuring robustness. Sensitivity analyses including MR-PRESSO, leave-one-out, MR-Egger, and Cochran Q tests were conducted to validate the IVW results, enhancing the reliability of our findings. Second, by employing germline genetic variation as an instrumental variable for exposure to effectively avoid reverse causality and confounders while circumventing ethical risks, our study achieved random assignment.

However, our study is not without limitations. First, the distribution of EM DN (CD4–CD8–) T cells was analyzed using flow cytometry, limiting insights into the cell type-specific functional status. Second, the study population was exclusively of people of European origin, introducing potential ethnic bias and limiting the generalizability of the results. Third, the unavailability of raw data restricted our analysis to only estimate approximate causality between variables but not detailed causality assessments. Finally, while our study makes preliminary assessments of causality between GM, immune cells, and CHD, the underlying biological mechanisms remain inadequately understood. Future studies are necessary to further elucidate and confirm the relationships between GM, immune cells, and CHD.

## 5 Conclusion

In summary, we extensively validated the relationship between GM, immune cells, and CHD. Specifically, we found that an increase in the intestinal bacterium g__Desulfovibrio.s__Desulfovibrio_piger enhances the levels of EM DN (CD4–CD8–) %T cells, an immune cell subtype. This increase in EM DN (CD4–CD8–) %T cells correspondingly reduced the risk of developing CHD. While these findings concerning intestinal flora and immune cells offer valuable insights for early diagnostics, prevention, and treatment strategies, further research is necessary to support these conclusions. Our results provide a foundation for potential strategies to reduce the incidence of CHD through the modulation of GM and the regulation of immune cells.

## Data availability statement

The data presented in the study are deposited in the BioStudies repository, accession number S-BSST1516.

## Ethics statement

Ethical approval was not required for the studies involving humans because the data required for this study were obtained from public databases and did not involve direct research on humans or animals.

## Author contributions

FY: Methodology, Writing – original draft, Software. HS: Methodology, Writing – original draft, Software. WT: Formal analysis, Writing – original draft. LL: Formal analysis, Writing – original draft. ZZ: Data curation, Writing – original draft. BO: Data curation, Writing – original draft. LZ: Data curation, Writing – original draft. GH: Funding acquisition, Writing – original draft, Writing – review & editing. WQ: Conceptualization, Writing – original draft, Writing – review & editing.
